# Antifibrotic Drugs Regulate the Expression of Epithelial Sodium Channels in the Lungs

**DOI:** 10.3390/arm94030030

**Published:** 2026-04-29

**Authors:** Toshiyuki Ito, Hajime Fujimoto, Masaaki Toda, Valeria Fridman D’Alessandro, Corina N. D’Alessandro-Gabazza, Yurie Kogue, Tatsuki Tsuruga, Tomohito Okano, Kazuki Furuhashi, Haruko Saiki, Atsushi Tomaru, Esteban C. Gabazza, Taro Yasuma, Tetsu Kobayashi

**Affiliations:** 1 Department of Pulmonary and Critical Care Medicine, Faculty and Graduate School of Medicine, Mie University, Edobashi 2-174, Tsu 514-8507, Mie, Japan; 2 Department of Immunology, Faculty and Graduate School of Medicine, Mie University, Edobashi 2-174, Tsu 514-8507, Mie, Japan; 3 Microbiome Research Center, Mie University, Edobashi 2-174, Tsu 514-8507, Mie, Japan

**Keywords:** pulmonary fibrosis, epithelial sodium channels, TGF-β1, pirfenidone, nintedanib

## Abstract

**Highlights:**

**What are the main findings?**

- TGF-β1 markedly suppresses epithelial sodium channel (ENaC) and CFTR expression in lung epithelial cells.
- Antifibrotic drugs nintedanib and pirfenidone prevent TGF-β1-induced downregulation of ENaC subunits in vitro and in vivo.

**What are the implications of the main findings?**

- Antifibrotic therapy preserves epithelial ion transport by restoring ENaC expression in the lung epithelium.
- Maintenance of epithelial sodium homeostasis may represent a previously unrecognized mechanism underlying antifibrotic efficacy in lung fibrosis.

**Abstract:**

Purpose: A high-salt extracellular environment promotes fibrosis in multiple organs by inducing oxidative stress, fibroblast activation, and extracellular matrix remodeling. In the lung, sodium accumulation may result from impaired epithelial ion transport. Transforming growth factor-β1 (TGF-β1), a key profibrotic cytokine, downregulates epithelial sodium and chloride channels, promoting sodium retention and fibrotic remodeling. This study investigated whether antifibrotic drugs can prevent TGF-β1-induced suppression of sodium channel expression in the lung epithelium. Methods: Human A549 alveolar epithelial cells and primary alveolar epithelial cells were cultured with or without TGF-β1 in the presence or absence of nintedanib or pirfenidone. Expression of epithelial sodium channel (ENaC) subunits (*SCNN1A*, *SCNN1B*, *SCNN1G*, *SCNN1D*) and *CFTR* was analyzed. In vivo, lung tissues from TGF-β1 transgenic mice and wild-type controls were examined following intranasal administration of pirfenidone. Results: TGF-β1 markedly reduced the expression of all ENaC subunits and *CFTR* in vitro. Nintedanib prevented suppression of *SCNN1A*, *SCNN1D*, and *SCNN1G*, whereas pirfenidone prevented suppression of *SCNN1A*, *SCNN1B*, and *SCNN1G*. In TGF-β1 transgenic mice, Scnn1a, Scnn1b, and Scnn1g expression was significantly decreased compared with wild-type controls. Pirfenidone administration dose-dependently restored expression of these ENaC subunits in vivo. Conclusions: Antifibrotic drugs partially prevent TGF-β1-induced suppression of epithelial sodium channels, preserving epithelial ion homeostasis. Restoration of ENaC expression may represent a novel mechanism by which antifibrotic therapy mitigates sodium-associated lung fibrosis.

## 1. Introduction

Fibrosis represents a pathological hallmark of chronic tissue injury, characterized by excessive extracellular matrix (ECM) deposition, fibroblast activation, and progressive architectural distortion across multiple organs. Mounting evidence indicates that high-salt environments promote fibrogenesis by inducing oxidative stress, disrupting ECM homeostasis, and activating profibrotic signaling pathways. Excessive salt intake accelerates liver fibrosis via reactive oxygen species (ROS)-mediated mechanisms, while elevated dietary salt disrupts mitochondrial calcium homeostasis, leading to hypertrophic skin scarring [1,2]. Similarly, in the gastrointestinal tract, dietary salt exacerbates intestinal fibrosis in colitis models by enhancing fibroblast activation [3]. Cardiac tissue exhibits comparable vulnerability to salt-induced remodeling, where overexpression of IGF-IIRα aggravates salt-induced apoptosis and myocardial fibrosis [4]. In peritoneal tissues, high-salt exposure amplifies both inflammatory and fibrotic responses [5]. In the kidney, excessive sodium chloride intake promotes renal fibrosis in aging mice, while dietary NaCl modulates bleomycin-induced lung fibrosis [6,7]. Furthermore, vascular fibrosis has been linked to salt-induced structural remodeling independent of changes in blood pressure [8]. Collectively, these findings underscore that excessive sodium exposure acts as a systemic profibrotic trigger, underscoring the need to regulate sodium intake to prevent pathological tissue remodeling across organ systems.

A growing body of research suggests that a high-salt microenvironment may result from the extracellular accumulation of osmotically inactive sodium within the interstitial space. Sodium ions can bind to negatively charged glycosaminoglycans (GAGs), such as heparan sulfate, chondroitin sulfate, dermatan sulfate, and keratan sulfate, which are abundantly expressed in fibrotic tissues [9,10]. This non-osmotic sodium storage mechanism alters local tonicity and cellular signaling within the extracellular matrix [11,12]. Importantly, transforming growth factor-beta 1 (TGF-β1), a master regulator of fibrosis, appears to contribute to this aberrant sodium retention. TGF-β1 has been shown to suppress Na^+^ and Cl^−^ transport across alveolar surfaces by downregulating epithelial sodium (ENaC) and chloride (CFTR) channels, thereby promoting extracellular sodium accumulation and indirectly facilitating fibrotic remodeling [13,14,15,16,17]. Consistent with these mechanisms, our previous studies demonstrated significantly elevated sodium concentrations in the fibrotic lung tissues of transgenic mice overexpressing TGF-β1 in pulmonary epithelium [18]. Notably, tissue sodium levels correlated negatively with ENaC expression but positively with ECM deposition and pro-inflammatory cytokine expression [18]. The detection of halophilic microbial species in fibrotic lung tissues further supports the hypothesis that the fibrotic matrix constitutes a saline microenvironment that may shape the pulmonary microbiome [19].

Despite significant advances, curative therapies for idiopathic pulmonary fibrosis (IPF) remain unavailable. The currently approved antifibrotic agents, nintedanib and pirfenidone, provide clinical benefits, primarily slowing the decline in forced vital capacity (FVC), but do not consistently improve survival [20]. Although the precise mechanisms of action of both agents remain incompletely understood, both agents have been proposed to modulate multiple signaling pathways, including TGF–β1-dependent profibrotic cascades [21]. In the present study, we hypothesize that nintedanib and pirfenidone may exert part of their antifibrotic efficacy by counteracting TGF-β1-mediated inhibition of epithelial sodium and chloride channels, thereby mitigating sodium accumulation and fibrosis progression in the lungs.

## 2. Materials and Methods

### 2.1. Reagents

The human lung epithelial cell line A549 was from the American Type Culture Collection (Manassas, VA, USA), Dulbecco’s Modified Eagle Medium (DMEM) from Sigma-Aldrich (Saint Louis, MO, USA), and fetal bovine serum (FBS) from Bio Whittaker (Walkersville, MD, USA). Primary human pulmonary alveolar epithelial cells (PAEpi) and the corresponding alveolar epithelial cell growth medium (AEpiCM) were obtained from ScienCell Research Laboratories (Carlsbad, CA, USA). L-glutamine, penicillin, and streptomycin were from Invitrogen (Carlsbad, CA, USA). Nintedanib was purchased from Cayman Chemical Co. (Ann Arbor, MI, USA). Pirfenidone was purchased from Tokyo Chemical Industry Co. (Tokyo, Japan) for the in vitro study and provided by Shionogi Co. (Osaka, Japan) for the in vivo study.

### 2.2. Cell Stimulation

A549 cells were cultured to subconfluence, washed, and then incubated in serum-free medium containing varying concentrations of nintedanib (0, 5, or 10 µM) or pirfenidone (0, 0.5, or 1 mg/mL) before stimulation with 10 ng/mL TGF-β1. The mRNA expression levels of the non-voltage-gated epithelial sodium channel subunits *SCNN1A*, *SCNN1B*, *SCNN1D*, and *SCNN1G* were subsequently quantified by RT–PCR. Primary human alveolar epithelial cells were similarly cultured to subconfluence, incubated in serum-free medium containing nintedanib (10 µM) or pirfenidone (1 mg/mL), and then stimulated with TGF-β1 for 24 h. The mRNA expression levels of *SCNN1A*, *SCNN1B*, and *SCNN1G* were subsequently quantified by RT–PCR. The concentrations of nintedanib and pirfenidone used in the in vitro experiments were selected to evaluate antifibrotic effects under controlled cell-culture conditions and were not derived by direct extrapolation from the in vivo mouse doses or from clinical dosing. This approach is consistent with previous mechanistic studies in lung tissue and cell systems, in which nintedanib has commonly been used at low micromolar concentrations and pirfenidone at millimolar concentrations [22,23].

A549 cells were used in most experiments as a practical, stable, and reproducible in vitro system for mechanistic analyses, as they retain several characteristics of alveolar epithelial type II-like cells [24,25]. Primary alveolar epithelial cells were used to validate key findings, but their limited capacity for expansion and tendency to undergo phenotypic changes or senescence during prolonged culture limited their use in more extensive experiments.

### 2.3. Evaluation of Protein Expression

Primary human pulmonary alveolar epithelial cells were cultured to confluence, serum-starved for 24 h, and then stimulated with human TGF-β1 in the presence or absence of pirfenidone (1 mg/mL) or nintedanib (10 μM) for an additional 24 h. After treatment, cells were washed twice with cold phosphate-buffered saline (PBS) and lysed in radioimmunoprecipitation assay (RIPA) buffer supplemented with protease and phosphatase inhibitors. Protein concentrations were measured using a bicinchoninic acid (BCA) Protein Assay Kit (Thermo Scientific, Rockford, IL, USA). Equal amounts of total protein (10 μg per lane) were separated by 10–20% SDS-polyacrylamide gel electrophoresis and transferred onto PVDF membranes. Membranes were blocked with 5% (*w*/*v*) skim milk in Tris-buffered saline containing 0.1% Tween-20 (TBS-T) for 1 h at room temperature and then incubated overnight at 4 °C with rabbit anti-ENaC-α antibody (GTX110436; GeneTex, Irvine, CA, USA; 1:1000) or anti-β-actin antibody (clone 13E5, cat. no. 4970; Cell Signaling Technology, Danvers, MA, USA; 1:4000). After three washes with TBS-T, membranes were incubated for 1 h at room temperature with a horseradish peroxidase-linked anti-rabbit IgG secondary antibody (cat. no. HAF008; R&D Systems, Minneapolis, MN, USA; 1:2000). Immunoreactive bands were visualized using an ImageQuant LAS 4000 mini (GE Healthcare Life Sciences, Uppsala, Sweden). Band intensities were quantified using ImageJ vs 1.54p software (NIH, Bethesda, MD, USA) and normalized to β-actin expression. The data shown are representative of two independent experiments.

### 2.4. Evaluation of Ion Channel Expression in a TGF-β1 Transgenic Mouse Model of Lung Fibrosis

A transgenic (TG) mouse line expressing the full-length human TGF-β1 gene under the control of the mouse surfactant protein C promoter was previously generated using a bacterial artificial chromosome construct on a C57BL/6 background [26]. These mice spontaneously develop progressive pulmonary fibrosis beginning at approximately six weeks of age. All mice were housed in a specific pathogen-free facility at Mie University under controlled conditions with a **12-h** light/dark cycle. Chest micro-computed tomography (mCT) imaging and genotyping of transgenic (TG) mice were conducted as previously described [18].

To evaluate the effect of pirfenidone on epithelial sodium channel gene expression in vivo, lung tissue samples from pirfenidone-treated TGF-β1 transgenic (TG) mice with established fibrosis were analyzed. Twelve 9-week-old male TG mice with CT-confirmed and matched fibrotic lesions were randomly assigned to two treatment groups: TGF-β1/PFD-16 (*n* = 6), receiving pirfenidone at 16 mg/kg, and TGF-β1/PFD-24 (*n* = 6), receiving pirfenidone at 24 mg/kg. Pirfenidone was administered by intranasal instillation twice daily for 21 days and once on day 22 before sacrifice. Age-matched wild-type (WT) mice (*n* = 6) treated with saline according to the same schedule served as controls. The in vivo doses were selected based on our pilot experimental experience in this model and were not derived by direct extrapolation from the in vitro concentrations. Because the therapeutic efficacy of inhaled pirfenidone against lung fibrosis in this model has been demonstrated previously [27], the present study was designed specifically to assess changes in ion transport-related gene expression in lung tissue. Accordingly, serial post-treatment CT imaging, additional fibrosis marker assessments, and histopathological or immunohistochemical evaluations were beyond the scope of the current study.

### 2.5. Gene Expression Analysis

Total RNA was extracted from cells or lung tissue using Sepasol RNA-I Super G (Nacalai Tesque Inc., Kyoto, Japan). Complementary DNA (cDNA) was synthesized from 2 µg of total RNA using oligo (dT) primers and ReverTra Ace Reverse Transcriptase (Toyobo Life Science Department, Osaka, Japan). Real-time quantitative PCR (qPCR) was performed using gene-specific primer sets listed in Supplementary Table S1, which includes the primer sequences, accession numbers, primer positions, melting temperatures, and expected amplicon sizes. Quantitative amplification was carried out using an Applied Biosystems StepOne Real-Time PCR System and PowerUp™ SYBR™ Green Master Mix (Applied Biosystems/Thermo Fisher Scientific, Waltham, MA, USA) according to the manufacturer’s instructions. The amplification conditions were as follows: initial denaturation at 95 °C for 10 min, followed by 40 cycles of denaturation at 95 °C for 15 s and annealing/extension at 60 °C for 1 min. Relative gene expression was analyzed using StepOne Software v2.1 (Applied Biosystems) and normalized to GAPDH (human) or Gapdh (mouse).

### 2.6. Statistical Analysis

Data are described as the mean ± standard deviation (SD) of the means unless otherwise stated. The statistical difference between two variables was assessed using a two-tailed unpaired *t*-test, and the difference between three or more variables was assessed using analysis of variance (ANOVA) with the Neuman-Keuls test for post hoc analysis. A *p*-value < 0.05 was considered statistically significant. We performed the statistical analysis using GraphPad Prism version 10.6.1 (GraphPad Software, Inc., San Diego, CA, USA).

## 3. Results

### 3.1. TGF-β1 Downregulates Sodium Channel mRNA Expression in Lung Epithelial Cells

**Figure 1:**
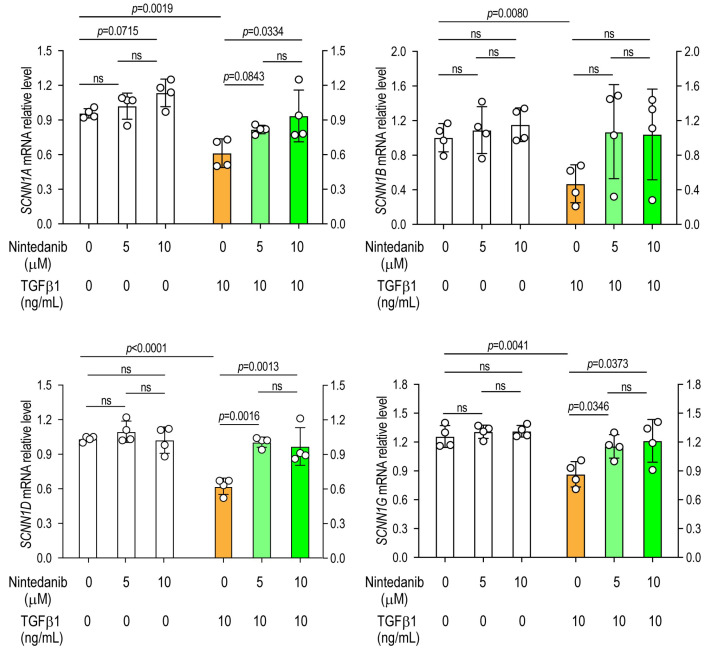
Nintedanib prevents TGF-β1-induced suppression of epithelial sodium channel expression. A549 alveolar epithelial cells were cultured for 24 h with or without TGF-β1 in the presence or absence of increasing concentrations of nintedanib. The mRNA expression levels of epithelial sodium channel (ENaC) subunits were quantified by RT-PCR. Data are presented as mean ± SD. *N* = 4 in each group. Representative results of two independent experiments are shown. Statistical analyses comparing TGF-β1-treated cells with untreated control cells were performed using a two-tailed unpaired *t*-test. Comparisons among different doses of antifibrotic agents were conducted using one-way analysis of variance (ANOVA), followed by the Newman–Keuls post hoc test. ns, not significant.

**Figure 2:**
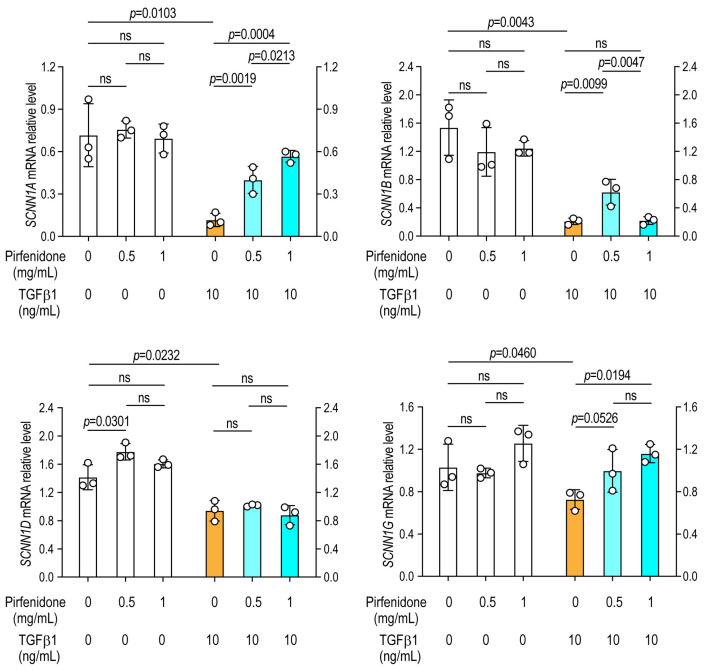
Pirfenidone prevents TGF-β1-induced suppression of epithelial sodium channel expression. A549 alveolar epithelial cells were cultured for 24 h with or without TGF-β1 in the presence or absence of increasing concentrations of pirfenidone. The mRNA expression levels of ENaC subunits were determined by RT-PCR. Data are expressed as mean ± SD. *N* = 3 in each group. Representative results of two independent experiments are shown. Statistical analyses comparing TGF-β1-treated cells with untreated control cells were performed using a two-tailed unpaired *t*-test. Comparisons among different doses of antifibrotic agents were conducted using one-way analysis of variance (ANOVA), followed by the Newman–Keuls post hoc test. ns, not significant.

A549 alveolar epithelial cells were cultured for 24 h in the presence or absence of TGFβ1 and various concentrations of nintedanib or pirfenidone, followed by the extraction of total RNA to assess sodium and chloride channel gene expression by RT-PCR. Exposure to TGF-β1 resulted in a significant downregulation of *SCNN1A* (α-ENaC), *SCNN1B* (β-ENaC), *SCNN1D* (δ-ENaC), and *SCNN1G* (γ-ENaC) compared with saline-treated (vehicle control) cells, indicating that TGF-β1 suppresses epithelial sodium channel expression in lung epithelial cells (Figure 1 and Figure 2).

### 3.2. Nintedanib Prevents TGF-β1-Induced Suppression of Sodium Channel Expression

Treatment with nintedanib alone did not significantly alter the expression of any sodium channel subunits under basal conditions (Figure 1). However, in TGF-β1-stimulated cells, nintedanib markedly restored the expression of *SCNN1A*, *SCNN1D*, and *SCNN1G*. In contrast, the TGF-β1-mediated suppression of *SCNN1B* was not prevented by nintedanib treatment, suggesting subunit-specific responsiveness to antifibrotic modulation (Figure 1).

### 3.3. Pirfenidone Prevents the Downregulation of Sodium Channels

Under basal conditions, pirfenidone treatment led to a modest but statistically significant increase in *SCNN1D* expression, with no detectable changes in other sodium channel subunits (Figure 2). In TGF-β1-treated cells, pirfenidone significantly restored expression of *SCNN1A*, *SCNN1B*, and *SCNN1G*, whereas *SCNN1D* suppression persisted despite treatment (Figure 2). These findings suggest that pirfenidone exerts selective restorative effects on sodium channel gene expression following TGF-β1 exposure.

### 3.4. Antifibrotic Drugs Do Not Restore CFTR Expression

**Figure 3:**
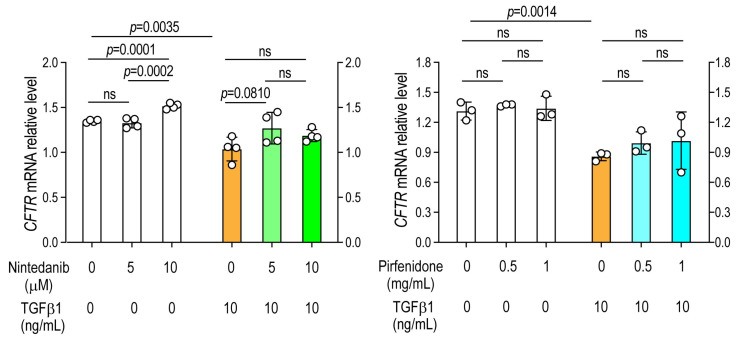
Antifibrotic treatment fails to rescue TGF-β1-mediated *CFTR* downregulation. A549 alveolar epithelial cells were cultured for 24 h with or without TGF-β1 in the presence or absence of increasing concentrations of pirfenidone or nintedanib. CFTR mRNA expression was analyzed by RT-PCR. Data are expressed as mean ± SD. *N* = 4 in the groups treated with nintedanib, and *n* = 3 in the groups treated with pirfenidone. Representative results of two independent experiments are shown. Statistical analyses comparing TGF-β1-treated cells with untreated control cells were performed using a two-tailed unpaired *t*-test. Comparisons among different doses of antifibrotic agents were conducted using one-way analysis of variance (ANOVA), followed by the Newman–Keuls post hoc test. ns, not significant.

Exposure to TGF-β1 significantly reduced *CFTR* expression compared with saline-treated controls. Neither nintedanib nor pirfenidone prevented the TGF-β1-induced downregulation of *CFTR*, indicating that these antifibrotic agents selectively modulate epithelial sodium channel (ENaC) expression but not chloride channel (*CFTR*) expression under profibrotic conditions (Figure 3).

### 3.5. Recovery of Sodium Channel Expression by Antifibrotic Drugs in Primary Alveolar Epithelial Cells

**Figure 4:**
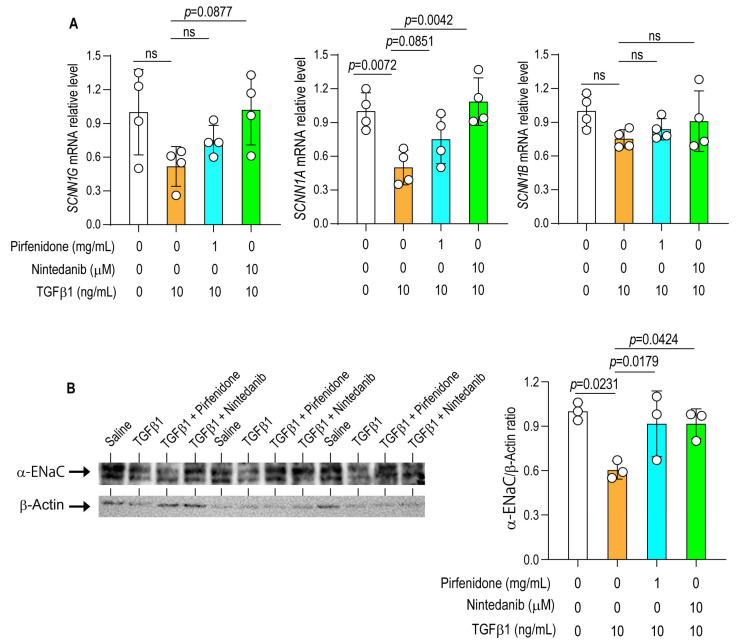
Antifibrotic agents prevent TGF-β1-induced suppression of epithelial sodium channel expression in primary alveolar epithelial cells. (**A**) Primary alveolar epithelial cells were cultured for 24 h with or without TGF-β1 in the presence or absence of pirfenidone or nintedanib. The mRNA expression levels of epithelial sodium channel (ENaC) subunits were determined by RT-PCR. *N* = 4 in each group. (**B**) Cells were cultured under the same conditions, and the protein expression of α-ENaC was evaluated by Western blotting. *N* = 3. Quantitative data are presented as the mean ± SD. Statistical analysis was conducted using one-way analysis of variance (ANOVA), followed by the Newman–Keuls post hoc test. ns, not significant.

The effects of antifibrotic drugs on TGF-β1-induced downregulation of sodium channel expression were further validated using primary alveolar epithelial cells. The relative mRNA expression level of *SCNN1A* was significantly reduced following TGF-β1 treatment compared with control cells (Figure 4A). TGF-β1 also decreased the mRNA relative expressions of *SCNN1B* and *SCNN1G*; however, these reductions did not reach statistical significance compared with controls.

Because the α-subunit of the epithelial sodium channel (*SCNN1A*) is essential for ENaC channel assembly and activity in alveolar epithelial cells, we also evaluated the effects of antifibrotic drugs on *SCNN1A* protein expression in primary alveolar epithelial cells by Western blot analysis. TGF-β1 markedly suppressed *SCNN1A* protein expression compared with control cells. Treatment with nintedanib and pirfenidone significantly increased *SCNN1A* protein levels compared with TGF-β1-treated cells (Figure 4B).

### 3.6. Pirfenidone Restores Epithelial Sodium Channel Expression In Vivo

**Figure 5:**
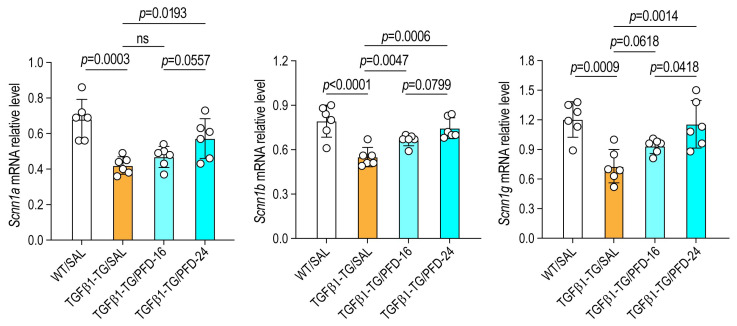
Pirfenidone restores epithelial sodium channel expression in vivo. Transgenic mice overexpressing TGF-β1 (TGF-β1 TG) were treated intranasally with pirfenidone at 16 mg/kg (TGF-β1/PFD-16; *n* = 6) or 24 mg/kg (TGF-β1/PFD-24; *n* = 6) twice daily for 21 days and once on day 22 before sacrifice. Wild-type (WT) mice (*n* = 6) served as controls. Lung mRNA expression of epithelial sodium channel subunits was analyzed by RT-PCR. Data are presented as mean ± SD. Statistical analysis was conducted using one-way analysis of variance (ANOVA), followed by the Newman–Keuls post hoc test. ns, not significant.

In TGF-β1 TG mice, the mRNA expression levels of *Scnn1a*, *Scnn1b*, and *Scnn1g* were significantly reduced compared with WT saline-treated controls. Administration of pirfenidone significantly prevented the TGF-β1-induced down-regulation of these genes (Figure 5). These findings indicate that pirfenidone exerts a restorative effect on epithelial sodium channel expression in vivo, counteracting the suppressive influence of TGF-β1 signaling.

## 4. Discussion

In this study, we demonstrate that antifibrotic agents restore ENaC expression suppressed by TGF-β1, both in vitro and in vivo. TGF-β1 markedly reduced ENaC subunit expression, whereas nintedanib and pirfenidone selectively prevented this effect. Consistent with these findings, pirfenidone administration significantly attenuated TGF-β1-induced suppression of ENaC gene expression in TGF-β1 transgenic mice with pulmonary fibrosis. Therefore, the principal novelty of the present study lies in identifying dysregulated epithelial sodium transport as a previously underappreciated component of profibrotic epithelial dysfunction and in showing that pirfenidone and nintedanib partially restore ENaC-related expression under profibrotic conditions.

Reduced ENaC expression in the lungs of TGF-β1 transgenic mice may have important implications for alveolar ion and fluid homeostasis. Previous studies have shown that TGF-β1 decreases αENaC expression, alters ENaC trafficking, and suppresses alveolar epithelial sodium and fluid transport, thereby impairing alveolar fluid clearance [13,14,16]. Evidence further suggests that lung fluid homeostasis is disturbed in idiopathic pulmonary fibrosis (IPF), primarily through alveolar epithelial barrier disruption, increased permeability, and altered epithelial ion transport, rather than through consistent overt pulmonary edema in stable disease [28]. In addition, single-cell RNA sequencing of lungs from patients with IPF has demonstrated altered expression of multiple epithelial sodium and chloride transporters, suggesting that transmembrane ion transport is broadly disrupted in fibrotic lungs [29]. Building on previous evidence implicating a high-salt microenvironment in tissue fibrosis, our findings further support the concept that dysregulated sodium transport contributes to the development and persistence of fibrotic tissue remodeling. Elevated extracellular sodium concentrations have been shown to enhance inflammation, oxidative stress, and fibroblast activation, thereby promoting excessive extracellular matrix deposition in multiple organs [3,6,12,30]. Moreover, sodium can accumulate in an osmotically inactive form within the extracellular matrix through electrostatic interactions with negatively charged glycosaminoglycans, thereby altering local ionic homeostasis and sustaining profibrotic signaling [12]. This concept is particularly relevant to pulmonary fibrosis, in which glycosaminoglycans are increased and structurally altered in the lung extracellular matrix [31,32,33]. Consistent with these mechanisms, our previous work demonstrated increased sodium content in fibrotic lungs from TGF-β1 transgenic mice, which correlated positively with extracellular matrix and cytokine expression and inversely with ENaC gene expression [18]. Although we did not directly assess lung fluid balance in the present study, these findings support the concept that TGF-β1-induced suppression of epithelial ion channels may contribute to disturbed fluid and salt homeostasis in pulmonary fibrosis.

Building on previous evidence implicating a high-salt environment in the pathogenesis of tissue fibrosis, our findings further support the concept that dysregulated sodium transport contributes to the development and persistence of fibrotic tissue remodeling. Elevated extracellular sodium concentrations have been shown to enhance inflammation, oxidative stress, and fibroblast activation, ultimately driving excessive ECM deposition across multiple organs [3,6,12,30]. Sodium can accumulate in an osmotically inactive form within the extracellular matrix through electrostatic interactions with negatively charged glycosaminoglycans, thereby altering local ionic balance and sustaining profibrotic signaling [12]. Consistent with these mechanisms, our previous work demonstrated that fibrotic lungs in TGF-β1 transgenic mice exhibit increased sodium content, which correlates positively with ECM and cytokine expression but inversely with ENaC gene levels [18]. Together with the present data, these observations suggest that TGF-β1-mediated suppression of ENaC expression promotes extracellular sodium retention, which, in turn, may contribute to or potentiate fibrotic remodeling through interconnected biophysical and signaling mechanisms.

Mechanistically, TGF-β1 impairs epithelial ion transport by reducing the transcription and surface abundance of ENaC and CFTR through Smad-dependent and Smad-independent signaling cascades [13,14,15,16,17]. Decreased ENaC expression lowers transepithelial sodium absorption, promoting extracellular sodium accumulation, altered osmotic gradients, and enhanced fibroblast responsiveness. The restoration of ENaC expression by antifibrotic drugs thus implies a homeostatic mechanism by which these therapies maintain epithelial barrier integrity and fluid homeostasis.

Nintedanib and pirfenidone are approved antifibrotic agents with partially overlapping but distinct mechanisms of action. Both modulate TGF-β-driven profibrotic pathways, including MAPK activation and oxidative stress signaling [34,35]. Against this background, the subunit-specific differences observed in ENaC restoration suggest that these agents may exert differential effects on transcriptional or post-transcriptional regulation within the epithelial ion transport network. The recovery of Scnn1a, Scnn1b, and Scnn1g expression in pirfenidone-treated TG mice further supports a direct epithelial effect of pirfenidone, potentially contributing to restoration of sodium transport under chronic fibrotic stress.

Although direct clinical evidence linking lung tissue sodium levels per se to disease severity or to response to pirfenidone or nintedanib in IPF has not yet been established, relevant indirect human evidence supports the biological plausibility of dysregulated epithelial sodium transport in IPF. In particular, reduced expression of neural precursor cell expressed, developmentally down-regulated 4-2 (NEDD4-2), an important regulator of ENaC, has been reported in IPF lungs, and experimental deletion of Nedd4-2 in mice increases ENaC activity and promotes fibrotic remodeling [36,37,38]. Importantly, these findings primarily implicate altered ENaC regulation and sodium transport activity rather than uniformly increased ENaC transcript expression across all settings [36,37,38]. Therefore, our results are best interpreted as indicating that antifibrotic treatment may help preserve epithelial ion-transport-related gene expression under profibrotic conditions, within the broader context of epithelial sodium transport dysregulation in fibrosis.

The clinical relevance of the in vitro drug concentrations used in this study also warrants careful consideration. Because direct human lung tissue concentrations of pirfenidone and nintedanib have not been well established, comparison with clinically observed plasma concentrations may provide the most practical reference. Reported plasma Cmax values are approximately 39.7 ng/mL (83 nM) for nintedanib at 150 mg twice daily and 11.85 µg/mL (64 µM) for pirfenidone at 801 mg three times daily [39]. In the present study, the concentrations used in vitro were higher than the typical circulating levels reported in pharmacokinetic studies [39,40,41]. Therefore, the clinical relevance of the in vitro concentrations should be interpreted with caution. It is worth noting, however, that drug concentrations in target tissues may exceed those in the systemic circulation. For example, nintedanib has been reported to distribute extensively into lung tissue and to accumulate there over time [42]. In summary, although our results support an additional potential mechanism of action of these antifibrotic agents, the magnitude of the in vitro effects observed in the present study should not be directly equated with clinical responses.

Neither antifibrotic agent restored CFTR expression in our model, indicating that chloride transport remains impaired despite partial recovery of ENaC. Because alveolar fluid clearance depends on coordinated transepithelial sodium and chloride transport, incomplete recovery of CFTR is likely to limit the functional benefit of ENaC restoration alone [43]. CFTR and ENaC are functionally interconnected epithelial ion channels, and their balanced activity is important for maintaining appropriate epithelial surface hydration and fluid homeostasis [44]. Accordingly, restoration of ENaC expression in the setting of persistent CFTR suppression may permit only partial normalization of alveolar epithelial fluid handling [43]. This finding may also reflect CFTR’s relative resistance to recovery under profibrotic conditions, as TGF-β has been shown to suppress CFTR expression and function at both the mRNA and protein levels in epithelial cells [45,46]. In addition, TGF-β-dependent CFTR suppression has been linked to altered epithelial homeostasis and may amplify maladaptive remodeling responses [46,47]. Persistent loss of CFTR activity may therefore contribute not only to impaired chloride transport, but also to ongoing epithelial dysfunction despite partial correction of sodium channel expression. Clinically, this persistent CFTR suppression may help explain why partial correction of epithelial ion transport does not necessarily translate into full restoration of alveolar homeostasis, and may contribute to continued vulnerability to impaired fluid clearance and epithelial dysfunction in fibrotic lungs. From a therapeutic perspective, these results suggest that recovery of ENaC alone may be insufficient for full restoration of alveolar epithelial function, and that strategies capable of preserving or restoring both ENaC- and CFTR-dependent ion transport may be required to achieve more complete epithelial homeostasis in pulmonary fibrosis.

Although the precise mechanism by which antifibrotic therapy restored ENaC expression was not examined in the present study, several plausible pathways may be considered. TGF-β is known to suppress alveolar epithelial sodium transport by reducing αENaC expression and alveolar vectorial sodium and fluid transport through an ERK1/2-dependent pathway, and it can also acutely promote ENaC internalization from the epithelial surface [13,16,48]. Therefore, one possible explanation is that nintedanib and pirfenidone partially restored ENaC expression by attenuating upstream TGF-β-driven signaling events that normally reduce ENaC abundance and membrane availability [49,50,51]. This interpretation is biologically plausible because nintedanib has been shown to inhibit TGF-β/Smad-associated epithelial responses in A549 cells and to suppress profibrotic signaling in experimental fibrosis models [50,51]. Pirfenidone has likewise been reported to interfere with TGF-β-related profibrotic signaling and oxidative stress pathways, both of which are relevant to epithelial dysfunction in fibrosis [49]. In addition, restoration of ENaC may involve post-transcriptional regulation, because ENaC surface expression is strongly controlled by the SGK1–Nedd4-2 axis, which regulates channel ubiquitination and endocytosis [52,53,54]. Thus, the subunit-specific recovery observed in our study may reflect combined effects on TGF-β-dependent transcriptional repression, ERK/Smad signaling, oxidative stress, and ENaC trafficking machinery rather than a single dominant mechanism [13,16,49,54]. Further studies will be needed to determine which of these pathways is primarily responsible for restoring ENaC expression in antifibrotic drug-treated epithelial cells.

This study has several limitations. First, although ENaC expression was assessed at both the gene and protein levels, the study relied predominantly on mRNA expression data, and the protein analysis was limited in scope and should be interpreted cautiously. In addition, direct functional assays, such as Ussing chamber measurements or patch-clamp analysis, were not performed, and alveolar fluid clearance was not directly evaluated. Therefore, the functional significance of ENaC restoration could not be fully established. In addition, lung tissue sodium concentrations after antifibrotic treatment were not measured, and the persistent suppression of CFTR suggests that recovery of epithelial ion transport may have remained incomplete despite partial restoration of ENaC. Second, the molecular mechanisms underlying the differential regulation of individual ENaC subunits remain incompletely understood. Moreover, many of the mechanistic experiments were performed in A549 cells. Although this cell line is widely used and experimentally convenient, it does not fully recapitulate the biology of normal primary alveolar epithelial cells. Third, although both pirfenidone and nintedanib were examined in vitro, only pirfenidone was evaluated in vivo. Therefore, the in vivo relevance of the findings cannot be considered equivalent for the two antifibrotic agents, and further in vivo studies of nintedanib are needed to establish its physiological and translational relevance. Fourth, because the in vitro experiments were limited to **24-h** TGF-β1 stimulation, they primarily model an early profibrotic response. In chronic fibrotic disease, persistent exposure to TGF-β and related stimuli may additionally influence ENaC-related pathways through sustained epithelial reprogramming, altered channel trafficking and activity, and long-term remodeling of epithelial identity [16,55,56]. Finally, the in vivo analysis was limited to ion transport-related gene expression in lung tissue. Accordingly, serial post-treatment CT imaging, additional fibrosis marker measurements, and histopathological or immunohistochemical confirmation of the corresponding protein changes were not included in the present study.

## 5. Conclusions

In summary, TGF-β1 suppresses epithelial sodium channel (ENaC) expression in lung epithelial cells, a mechanism that may contribute to sodium accumulation and fibrotic remodeling in the lung. In this study, the antifibrotic agents nintedanib and pirfenidone partially prevented TGF-β1-induced ENaC downregulation in vitro, while pirfenidone restored ENaC expression in vivo in TGF-β1 transgenic mice. These findings suggest that preservation of epithelial sodium transport may represent an additional mechanism through which antifibrotic therapies help maintain epithelial homeostasis and mitigate fibrotic remodeling, although further studies are needed to confirm the functional and in vivo significance of this pathway.

## Data Availability

The raw data supporting the findings of this study are available in Zenodo at https://doi.org/10.5281/zenodo.18464012.

## Supplementary Materials

The following supporting information can be downloaded at: https://www.mdpi.com/article/10.3390/arm94030030/s1, Table S1: Primers for RT-PCR.
