# Supplementary material for: Antifibrotic Drugs Regulate the Expression of Epithelial Sodium Channels in the Lungs
Source: Adv Respir Med. 2026 Apr 29;94(3):30. doi: 10.3390/arm94030030 (PMC13214485; doi:10.3390/arm94030030)
Supplement: Supplementary file 1 [file arm-94-00030-s001.zip › arm-4226821-supplementary.pdf]

Supplementary Information

Antifibrotic Drugs Regulate the Expression of Epithelial Sodium Channels in the Lungs

Toshiyuki Ito, Hajime Fujimoto, Masaaki Toda, Valeria Fridman D'Alessandro, Corina N. D'Alessandro-Gabazza, Yurie Kogue, Tatsuki Tsuruga, Tomohito Okano, Kazuki Furuhashi, Haruko Saiki, Atsushi Tomaru, Esteban C. Gabazza, Taro Yasuma, and Tetsu Kobayashi.

Supplementary Table 1. Primers for RT-PCR

Human genes	Direction	Sequence (5' -> 3')	Length	Tm	Reference	Position	Product
GAPDH	Forward	GGAGCGAGATCCCTCCAAAAT	21	61.6	NM_001256799	108-128	197
	Reverse	GGCTGTTGTCATACTTCTCATGG	23	60.9		304-282	
SCNN1A	Forward	GCAGACGCTCTTTGACCTGTA	21	62.1	NM_001159575	534-554	242
	Reverse	AGTCCGATTTGTTCTGGTTGC	21	60.8		775-755	
SCNN1B	Forward	CCTGCCTATTCGGAGCTGAG	20	61.8	NM_000336	743-762	184
	Reverse	AAGGGGACGTAGTCTTCCTGG	21	62.7		926-906	
SCNN1G	Forward	AAAGCAGCCTAGATTCTCCCA	21	60.6	NM_001039	447-467	114
	Reverse	ATGATGCTACCGCCGACTTTC	21	62.5		560-540	
SCNN1D	Forward	ACTCTCTGGCCCGATACAGG	20	62.6	NM_00113041	246-265	187
	Reverse	CCATTCTCCCGTCCATGCTTC	21	62.8		432-412	
Mouse genes							
Gapdh	Forward	TGGCCTTCCGTGTTCCTAC	19	61.3	NM_008084	686-704	178
	Reverse	GAGTTGCTGTTGAAGTCGCA	20	60.9		863-844	
Scnn1a	Forward	TACTTCAGCTACCCCGTGAGT	21	62.6	NM_011324	403-423	153
	Reverse	AAAAAGCGTCTGTTCCGTGAT	21	64.6		555-535	
			23				
Scnn1b	Forward	TACCTTGCGGAACTTCACCAG	21	66.1	NM_011325	603-623	138
	Reverse	CAAGCTAGGATTATGCGATCAGG	23	60.6		740-718	
Scnn1g	Forward	GCACCGACCATTAAGGACCTG	21	62.7	NM_011326	64-84	118
	Reverse	GCGTGAACGCAATCCACAAC	20	62.8		181-162	

Data are expressed as mean ± SD. *CFTR*/*Cftr*, cystic fibrosis transmembrane conductance regulator; *SCNN1A*/*Scnn1a*, *SCNN1B*/*Scnn1b*, and *SCNN1G*/*Scnn1g*, alpha, beta, and gamma subunits of epithelial sodium channel 1, respectively; *GAPDH*/*Gapdh*, glyceraldehyde-3-phosphate dehydrogenase.
